# Isolation and Characterization of Two *Klebsiella pneumoniae* Phages Encoding Divergent Depolymerases

**DOI:** 10.3390/ijms21093160

**Published:** 2020-04-30

**Authors:** Pilar Domingo-Calap, Beatriz Beamud, Lucas Mora-Quilis, Fernando González-Candelas, Rafael Sanjuán

**Affiliations:** 1 Institute for Integrative Systems Biology, I^2^SysBio, Universitat de València-CSIC, 46980 Paterna, Spain; lucas.mora@uv.es (L.M.-Q.); fernando.gonzalez@uv.es (F.G.-C.); rafael.sanjuan@uv.es (R.S.); 2 Department of Genetics, Universitat de València, 46980 Paterna, Spain; 3 FISABIO-Salud Pública, Generalitat Valenciana, 46020 Valencia, Spain; beatriz.beamud@uv.es; 4 CIBER in Epidemiology and Public Health, 46020 Valencia, Spain

**Keywords:** *Klebsiella pneumoniae*, bacteriophage, phage therapy, wide infection range

## Abstract

The emergence of multidrug-resistant bacteria is a major global health concern. The search for new therapies has brought bacteriophages into the spotlight, and new phages are being described as possible therapeutic agents. Among the bacteria that are most extensively resistant to current antibiotics is *Klebsiella pneumoniae*, whose hypervariable extracellular capsule makes treatment particularly difficult. Here, we describe two new *K. pneumoniae* phages, *πVLC5* and *πVLC6*, isolated from environmental samples. These phages belong to the genus *Drulisvirus* within the family *Podoviridae*. Both phages encode a similar tail spike protein with putative depolymerase activity, which is shared among other related phages and probably determines their ability to specifically infect *K. pneumoniae* capsular types K22 and K37. In addition, we found that phage *πVLC6* also infects capsular type K13 and is capable of striping the capsules of *K. pneumoniae* KL2 and KL3, although the phage was not infectious in these two strains. Genome sequence analysis suggested that the extended tropism of phage *πVLC6* is conferred by a second, divergent depolymerase. Phage *πVLC5* encodes yet another putative depolymerase, but we found no activity of this phage against capsular types other than K22 and K37, after testing a panel of 77 reference strains. Overall, our results confirm that most phages productively infected one or few *Klebsiella* capsular types. This constitutes an important challenge for clinical applications.

## 1. Introduction 

*Klebsiella pneumoniae* is an encapsulated Gram-negative bacterium that can be found as a free-living organism, in natural environments. In addition, *K. pneumoniae* is an opportunistic pathogen causing infections mainly in the lungs and urinary tract, although meningitis and sepsis can occur in severe cases. Due to extensive antibiotic use and favorable transmission opportunities, multidrug-resistant *K. pneumoniae* strains, particularly carbapenem-resistant strains [1,2], have spread in hospitals and the community. *K. pneumoniae* belongs to the ESKAPE priority group defined by the WHO, which also includes *Enterococcus faecium, Staphylococcus aureus, Acinetobacter baumannii, Pseudomonas aeruginosa*, and *Enterobacter* spp. [3]. Thus, *K. pneumoniae* is considered to be an important target for the development of new therapies. 

Bacteriophages (phages) provide an interesting therapeutic alternative, since they infect bacteria in a specific manner, avoiding dysbiosis [4]. However, in the case of highly variable bacteria such as *K. pneumoniae*, the use of phages becomes challenging. It has been proposed that phage infectivity is strongly determined by the bacterial capsular type [5], making it difficult to treat a wide range of *K. pneumoniae* strains, since most of the phages described so far are specific to one or few capsular types. Based on this, the use of phage cocktails has been suggested, in which the interactions between phages can generate synergies that improve bacterial lysis and limit the emergence of resistances [6].

Due to the high heterogeneity of *K. pneumoniae*, it often becomes difficult to determine the virulence factors of this species [7]. Nevertheless, the capsule of *K. pneumoniae* is one such virulence factor, and hypervirulence has been associated with differences in the expression of genes involved in the capsule, or with modified capsule phenotypes [8]. Most *K. pneumoniae* phages have been found to encode depolymerases, which are enzymes capable of digesting the exopolysaccharide capsule by cleaving glycosidic linkages [9,10]. Disruption or capsule loss makes bacteria susceptible to phage infection or antibiotic treatment, suggesting a therapeutic potential for phage depolymerases [5,11,12,13,14]. Although most described phages encode a single depolymerase and, as a result, infect one or few capsular types, some phages have been reported to encode multiple depolymerases. To date, the most salient case is that of phage ØK64-1, which encodes nine different depolymerases [15]. Overall, a limited number of enzymes in active particular capsular types have been described [16]. In addition to their potential therapeutic applications, phage-borne depolymerases have been suggested for capsular typing [17]. For these reasons, discovering new *Klebsiella* phage depolymerases against distinct capsular types is an interesting topic with potential biomedical applications.

Recently, our group has isolated and characterized four new phages of *K. pneumoniae* (*πVLC1* to *πVLC4*) from environmental samples in Valencia, Spain [18]. All of these were closely related phylogenetically, belonged to the genus *Drulisvirus*, encoded only one depolymerase, and showed a very narrow capsular type range. Using a similar protocol, here we isolated two new phages with high lytic activity, which we named *πVLC5* and *πVLC6*. As opposed to our previously described phages, these new isolates were capable of infecting several reference capsular types of *Klebsiella* spp. Whole genome analysis revealed that *πVLC5* and *πVLC6* encoded divergent depolymerases, potentially explaining their host tropisms.

## 2. Results

### 2.1. Isolation and Phenotypic Characterization of Two New Klebsiella Pneumoniae Phages

**Figure 1:**
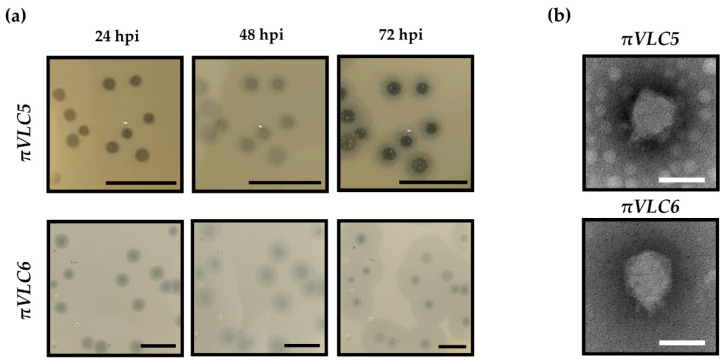
Initial characterization of *Klebsiella* phages *πVLC5* and *πVLC6*. (**a**) Plaque assays of the two phages. Plaques were allowed to develop in soft agar media at 37 °C (hpi: hours post inoculation). Notice that haloes around plaques kept expanding even though the lysis did not proceed further, suggesting passive diffusion of depolymerase activity. Scale bar: 1 cm. (**b**) Transmission electron micrographs of the two novel *K. pneumoniae* phages. Scale bar: 50 nm.

Phage *πVLC5* was obtained from a water sample in an irrigation ditch near a wastewater treatment plant, whereas phage *πVLC6* was isolated from a river, near its outflow to the sea, in an area where waste discharged by nearby villages accumulates. Our initial screening was carried out using a multidrug-resistant clinical isolate of *K. pneumoniae* as host, which belongs to capsular type K22. Once plaques were observed, they were further isolated and plaque-purified. Amplification of the phages in liquid medium was carried out to obtain a high-titer lysate and to proceed with detailed phage characterization. Plaque assays showed that the amplified phage *πVLC5* reached a titer of 2.4 × 10^11^ plaque forming units (PFU) per mL, while *πVLC6* reached 1.0 × 10^10^ PFU/mL. For both phages, large plaques were observed, which were surrounded by a halo that is often interpreted as an indicator of depolymerase-mediated digestion of bacterial capsules (Figure 1a). Haloes are formed because depolymerases can diffuse and degrade capsules in nearby bacteria even if lysis does not occur in these areas. This has been previously shown in other *Klebsiella* spp. [18] and in phages infecting other encapsulated bacteria, such as *Escherichia coli* or *Acinetobacter baumannii* [19,20]. The haloes of phage *πVLC6* were wider than those of *πVLC5*, whereas lysed areas were larger for *πVLC5*, suggesting that these were two distinct phages. Transmission electron microscopy micrographs obtained from high-titer lysates showed that the virion morphology of the two phages was similar and typical of podoviruses, with an icosahedral head of approximately 50 nm and a small tail of about 10 nm (Figure 1b).

### 2.2. Genome Sequencing and Comparative Genomics

**Figure 2:**
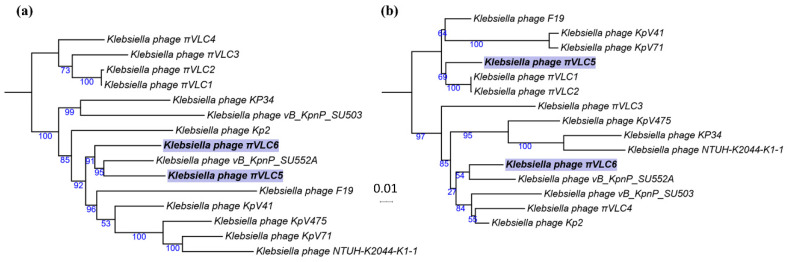
Maximum likelihood (ML) phylogenetic tree of the *Drulisvirus* genus. (**a**) Whole-genome and (**b**) RNA polymerase. Numbers indicate bootstrap support values (1000 pseudo-replicates). The two new phages are highlighted in purple. The scale bar represents 0.01 sequence divergence (percent nucleotide substitutions).

Genome sequencing of *πVLC5* yielded a single contig of 44,932 bp (54.3% G+C content) containing 58 putative coding sequences (CDSs). For phage *πVLC6*, a single contig was also obtained with 44,294 bp (54.2% G+C content) and 60 CDSs. The two phages showed a pairwise nucleotide identity of 92.5%. BLAST analysis showed that the two phages belonged to the family *Podoviridae*, genus *Drulisvirus*. To verify this, we obtained a multiple sequence alignment of all publicly available classified drulisviruses (n = 9), including the four additional genomes recently published by our group [18]. *Klebsiella* phages *πVLC5* and *πVLC6* were found to be collinear and similar to the rest of drulisviruses. To determine the evolutionary relationships among these phages, maximum likelihood (ML) phylogenies of whole-genome and RNA polymerase sequences were built. The whole-genome tree showed that *Klebsiella* phages *πVLC5* and *πVLC6* were members of a well-supported cluster within drulisviruses, but that πVLC5 was closer to the *Klebsiella* phage *Vb_KpnP_SU552A* [21] (Figure 2a). According to this phylogeny, both phages were phylogenetically separated from the previously described *πVLC1-4* phages. Yet, the RNA polymerase tree was not consistent with the whole genome tree and suggested a closer relationship between *πVLC5* and the previously described phages *πVLC1* and *πVLC2* (Figure 2b). 

### 2.3. Functional Annotation of K. pneumoniae Phages πVLC5 and πVLC6 

**Figure 3:**
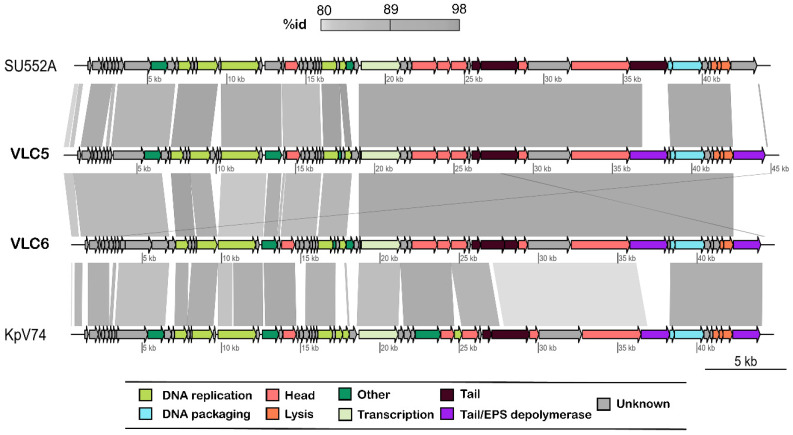
Genome alignment of the *Klebsiella* phages *πVLC5* and *πVLC6* with the previously characterized *Klebsiella* phages *vB_KpnP_SU552A* (closest relative) and *vB_KpnP_KpV7*4. Arrows represent coding sequences (CDSs). The different shades of gray represent different identity levels. The figure was obtained using genoPlotR [23].

Twenty-four conserved proteins were found among drulisviruses, including *Klebsiella* phages *πVLC5* and *πVLC6*. These included proteins involved in DNA replication (DNA polymerase and DNA-dependent RNA polymerase, 5’-3’ exonuclease, helicase, and primase), DNA packaging (DNA maturase A and B), head proteins (tegument, collar, capsid, internal virion, and core proteins), tail tubular proteins (A and B), enzymatic activities (phosphoesterase and peptidase), lysis proteins (spanin and holin), and additional, hypothetical proteins. Most differences were observed in the tail fiber proteins, where similarity dropped drastically between phages *πVLC5*, *πVLC6*, and the rest of drulisviruses, with a few exceptions (Figure 3, Table S1). Both phages contained two putative tail fiber proteins, with the second located after the lysis cassette, as previously observed [22]. 

The first tail-fiber protein was completely shared between the *πVLC5* and *πVLC6* phages (ORF49 and ORF51, respectively) and had a high structural similarity to the tail spike protein of bacteriophage *phiAB6* (e-value 3.9 × 10^−33^). Hence, we annotated both ORFs as tail spikes. In addition, these two ORFs showed high sequence similarity with the tail fiber protein of the *Pantoea* bacteriophage *LIMElight* (e-values 6.0 × 10^−40^ and 4.0 × 10^−40^, respectively), available from the depolymerases database. These ORFs contained two sequence domains. Positions 13–140 matched significantly with bacteriophage *T7* tail fiber protein, whereas positions 308–610 matched with a pectin lyase domain (Table 1), where the depolymerase activity is expected to reside. 

Phage *πVLC5* encoded a second tail fiber/spike protein (ORF58) that also matched significantly with the sequences in the depolymerase database, the best match being with the tail fiber protein of the *Klebsiella* phage *KP32* (e-value 1.4 × 10^−55^). InterProScan5 revealed three different domains in this ORF—a pectin lyase, an endosialidase chaperone, and a DNA-binding domain. In this case, the closest protein structure determined by HHpred was the tail spike of an *Acinetobacter* phage (Table 1). In turn, the second tail fiber protein of phage *πVLC6* (ORF58) was similar to a putative pectate lyase of *Serratia* phage *phiMAM1*. Surprisingly, no protein domains were found with InterProScan5 for this ORF. However, the ORF matched significantly with the protein structure of bacteriophage *CBA120* tail spike (Table 1). 

### 2.4. Divergence of Putative Depolymerase Sequences of Klebsiella Phages πVLC5 and πVLC6

**Figure 4:**
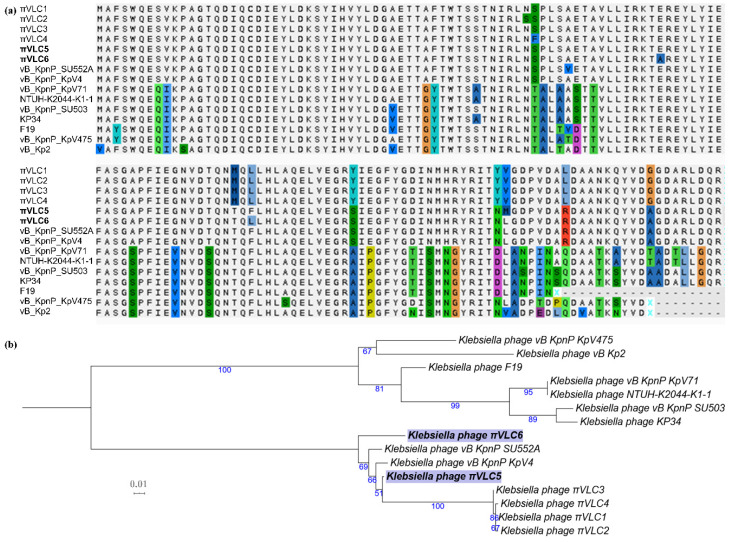
Conserved tail fibers/spikes of available drulisviruses (**a**). Amino acid sequence alignment of the conserved region of the tail fiber/spike. Variant amino acids are highlighted in different colors. (**b**). Nucleotide ML tree of the conserved region of the tail fiber/spike. Numbers indicate bootstrap values (1000 pseudo-replicates). The scale bar represents 0.01 sequence divergence (percent nucleotide substitutions).

To elucidate the degree of divergence and find putative orthologs for the different tail fibers/spikes of phages *πVLC5* and *πVLC6*, the above four ORFs were extracted and searched against a custom phage database with BLASTN. The tail fiber/spike shared among *πVLC5* and *πVLC6* (ORF49 and ORF51) was also present in the rest of *Drulisvirus* analyzed (Figure 4), including non-*Klebsiella* phages. However, only the first domain was highly conserved, whereas the second domain (where the putative enzymatic activity resides) was absent in all of these sequences except for the *πVLC* phages and partly in *Klebsiella* phage *KP32* (Table S1). The common region of the tail fiber was not restricted to this genus. In fact, it was similar to the tail fibers of *Przondovirus* and *Phikmvvirus* phages, all in the *Autographivirinae* subfamily (Table S1). Remarkably, the average nucleotide identity values dropped in this region, ranging from 70.7% to 99.8%, when compared to the rest of the genome (from 89.5% to 99.8%). Therefore, this protein seemed to be unique to the *πVLC* phages, given that the other phages only showed small regions of limited sequence identity. However, the ML phylogenetic tree of the core region of this ORF did not group the novel phages *πVLC5* and *πVLC6*, with the rest of *πVLC* phages (Figure 4).

Regarding the additional tail fiber/spikes of *πVLC5* and *πVLC6*, only three significant hits were found for the phage *πVLC5* ORF58 (Table S1). The best hit was a hypothetical protein of the *Klebsiella* phage *KOX1* with a sequence coverage of 51% and 68% nucleotide identity. This hit contained the pectin lyase domain. The second hit was the tail fiber protein of the *Klebsiella* phage *NJS1* (13% coverage and 67% identity). *Klebsiella* phages *KOX1* and *NJS1* belonged to the genus *Webervirus* of the family *Siphoviridae*. The last hit was almost non-significant (only 2% coverage but 89% of identity), with a protein of the *Vibrio* phage *1.164.O*. For phage *πVLC6* ORF58, we found three hits that covered the entire ORF, all from bacteriophages of the same genus, with levels of identity ranging from 94.8% to 95.7% (Table S1). 

### 2.5. Determination of the Host Range of the Two Klebsiella Pneumoniae Phages πVLC5 and πVLC6

**Figure 5:**
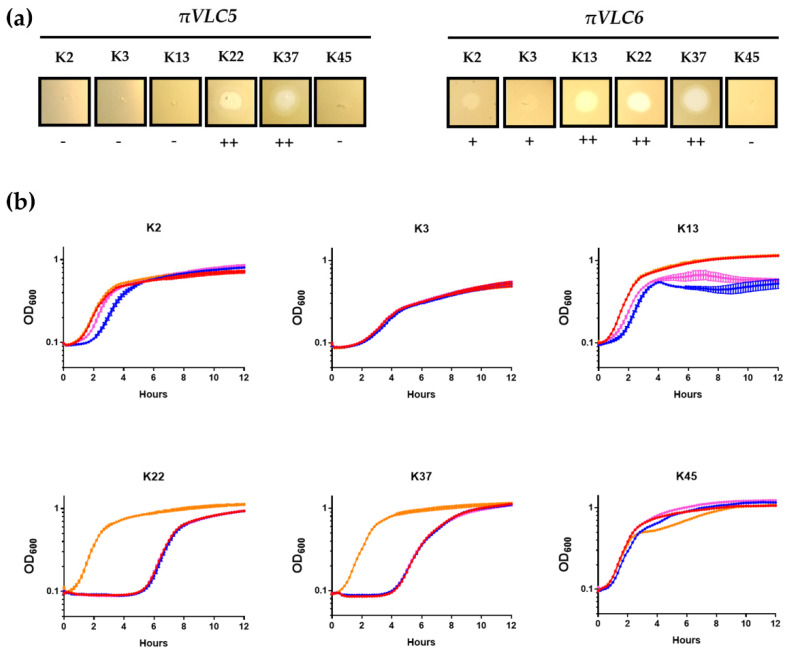
Lytic activity of *Klebsiella* phages *πVLC5* and *πVLC6* in different capsular types. (**a**) Spot tests of the two *K. pneumoniae* phages in K2, K3, K13, K22, K37, and K45 *Klebsiella* sp. reference strains. All 77 reference strains were assayed, but only those showing phage activity are shown, except for K45, which is included as an example of a non-susceptible strain. Plaques were allowed to develop in soft agar media overnight at 37 °C. ++: obvious lysis; +: halo-like turbidity reduction; -: no effect. (**b**) OD measurements of capsular types K2, K3, K13, K22, K37, and K45 *Klebsiella* spp. Orange—non-infected control; red*—πVLC5*; blue—*πVLC6*; pink—*πVLC5 + πVLC6.* Error bars indicate the SEM.

A panel of 77 reference strains of *Klebsiella* spp. purchased from the Statens Serum Institute (Copenhagen, Denmark) was used to test the infectivity of the newly discovered phages using spot tests in semi-solid medium and OD measurements in liquid cultures. Overall, the results of the spot tests were consistent with those obtained in liquid infections (Figure 5, Table S2). Phage *πVLC5* had a lytic ability against capsular types K22 and K37, whereas phage *πVLC6* lysed capsular types K22, K37, and K13 capsular types. Liquid cultures also showed that, for K22 and K37, phage-resistant bacteria emerged at around 5 h post-inoculation (hpi) and eventually reached densities similar to those of uninfected cultures at approximately 10 hpi, a process that could not be prevented using a cocktail of the two phages. In K13, *πVLC6*-mediated bacterial lysis occurred less efficiently but, on the other hand, the OD values remained lower than those of uninfected cultures even at 12 hpi. For K2 and, to a lesser extent, K3, spot tests revealed that phage *πVLC6* produced a slight reduction in the turbidity of the bacterial lawns. Liquid infections also indicated a slight growth delay in K2 cultures inoculated with *πVLC6*.

In bacterial strains where spot tests revealed an effect of the phage, phage progeny production was evaluated by inoculating low-density cultures (OD ca. 0.05) with ca. 10^5^ PFU (as determined in K22 plaque assays). At 4 hpi, phage titration showed that *πVLC5* and *πVLC6* did not produce any progeny in K2 and K3 cultures, since the endpoint titers were similar or lower than those at inoculation. The absence of phage progeny was also verified in semi-solid cultures inoculated by the spot test method. In contrast, K22 and K37 were productively infected by both phages, yielding >10^8^ PFU/mL of *πVLC5* and >10^9^ PFU/mL of *πVLC6*. In addition, phage *πVLC6* was able to produce >10^9^ PFU/mL in K13, as opposed to phage *πVLC5*.

### 2.6. Depolymerase Activity of Phage πVLC6

**Figure 6:**
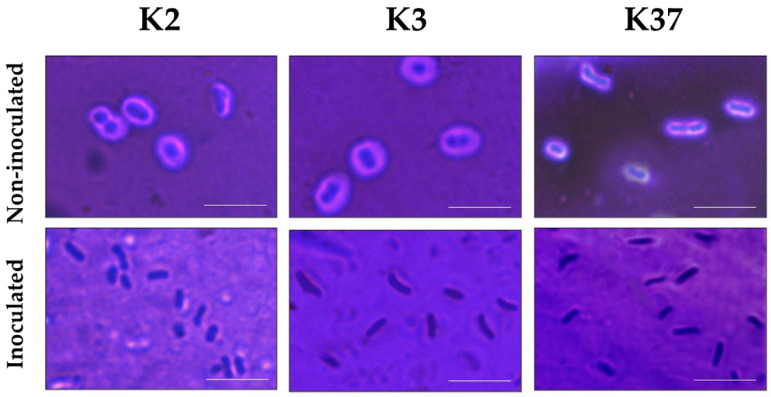
*Klebsiella* phage *πVLC6* depolymerase activity against K2 and K3 strains. Cultures were doubly stained with nigrosin and crystal violet. Capsules appeared as non-stained areas around cells. Inoculation with phage *πVLC6* produced a nearly complete stripping of the capsules. K37 strain was used as a positive control. Scale bar: 5 µm.

Phage *πVLC6* did not infect the reference strains of capsular types K2 and K3, as indicated by the absence of phage progeny. However, *πVLC6* did produce a halo in K3 lawns (and less obviously, also in K2 lawns) and slightly retarded the growth of K2 cultures. We hypothesized that this effect might be due to the ability of a *πVLC6* depolymerase to digest the K2 and K3 capsules. This depolymerase activity could be encoded by ORF58, which showed no homologous gene in *πVLC5*. To test this, we sampled the halo formed by *πVLC6* in K2 and K3 lawns for microscopy. For comparison, we also analyzed regions of the plate where no phage was added. These samples were stained with crystal violet to visualize bacteria and with nigrosin to provide background contrast. In non-inoculated K2 and K3 bacteria, capsules were intact and easily observable as non-stained areas around each cell (Figure 6). In contrast, capsules were obviously absent in K2 and K3 bacteria taken from the halo regions of the cultures inoculated with phage *πVLC6*. Our results confirmed the degradation of the exopolysaccharide capsule by the action of a depolymerase encoded by *πVLC6,* despite the inability of the phage to infect and lyse these strains.

### 2.7. Structural Comparison of K Antigens 

**Figure 7:**
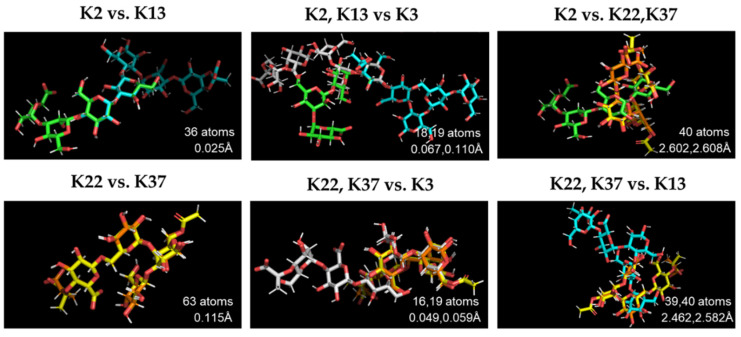
Structural superposition of *Klebsiella* K antigens K2, K3, K13, K22, and K37. The number of atoms that align between the pair-wise structures and the RMSD values are indicated. Green—K2 (85 atoms), white—K3 (112 atoms), blue—K13 (112 atoms), yellow—K22 (98 atoms), and orange—K37 (93 atoms).

Host-range experiments revealed that both phages *πVLC5* and *πVLC6* were able to infect capsular types K22 and K37. Additionally, phage *πVLC6* could infect K13 and digest K2 and K3 capsules. As depolymerases are thought to be antigenically specific, we wanted to determine whether these five capsular types shared some structural backbone that could make them recognizable by common enzymes. The *K. pneumoniae* capsule is a polysaccharide matrix of repeated units with an acidic component. K22 and K37 have the same sequence of sugars, with the exception of the presence of OAc substituents in K22 [24]. Approximately 66% of the atoms of K22 and K37 align properly, with low values of RMSD (0.115 Å) as expected (Figure 7). K2 and K13 shared the units of glucose, mannose, and glucuronic acid [25,26]. This could be observed in the overlapping of 36 atoms in the structural pairwise alignment with an RMSD value of 0.025 Å. The RMSD values between these two capsular types, K2 and K13, and those described above, K22 and K37, were much higher (>2.5 Å), showing a poor overlap of their molecular structures. Interestingly, antigen K3 overlapped partially with both clusters (K22/K37 and K2/K13), with RMSD ranging from 0.049 Å to 0.110 Å. The repeated units of the K3 structure were made of pyruvic acetal-bearing pentasaccharide containing galactose, mannose, and glucuronic acid [27]. Thus, K3 shared the sugars mannose with K2/K13 and galactose with K22/K37. 

## 3. Discussion 

The *K. pneumoniae* exopolysaccharide capsule is a major virulence factor and a source of diversity that complicates treatment [28]. Still relatively little is known about the diversity of *Klebsiella* sp. phages and the molecular mechanisms allowing infection of different capsular types. Although previous studies have proposed that depolymerases play a key role in the recognition and entry of phages in these bacteria, additional data are needed to establish more general patterns. Recently, our group has phenotypically and molecularly characterized four new *Drulisviruses* infecting capsular type K22 [18]. Despite the fact that this capsular type is not highly prevalent, it has been found among multi-drug-resistant clinical isolates. Moreover, *Klebsiella* capsular diversity is widely distributed, meaning that no single capsular type monopolizes a large fraction of clinical isolates and that, consequently, it would be necessary to target most types for efficiently combating this pathogen. Here, we have characterized two new phages capable of infecting not only K22, but also other capsular types. 

Phylogenetic analyses of whole and core genomes of the genus *Drulisvirus* group the new phages together, along with the previously described *Klebsiella* phage *Vb_KpnP_SU552A*. However, the phylogeny of the RNA polymerase does not support this grouping, placing *πVLC5* with other *πVLC* phages. These results, taken together with the lack of homology in one of the tail spikes, suggest a certain degree of mosaic-like genome structure for drulisviruses. Horizontal acquisition of tail spike genes seems especially relevant in the case of ORF58 of the *Klebsiella* phage *πVLC5* and ORF58 of *πVLC6*. The former presents a unique structure among the phage sequences described so far, as it is only distantly related to other phages from a different family. On the contrary, the second tail spike of *Klebsiella* phage *πVLC6* is also present in four other phages of the same genus, with a high level of identity. Regarding the shared tail spike present in all members of the genus *Drulisvirus* and some related phages (*Przondovirus* and *Phikmvvirus*), the enzymatic domain is only found in the *πVLC* phages, all of which share the ability to infect K22 capsular types. Interestingly, phage *πVLC5* was sampled in the same place as phages *πVLC1* and *πVLC2*, and these three phages seemed to form a monophyletic group according to the RNA polymerase phylogeny. However, they differed in their depolymerase domains and whole-genome phylogenetic position. This suggests that gene exchange events were involved in adaption to specific ecological niches [22].

Both phages described were capable of lysing capsular types K22 and K37. In addition, in both cases, lysis haloes were observed around plaques, presumably resulting from the action of their depolymerases [29]. *Klebsiella* strains presenting capsular types K22 and K37 shared most of the genes involved in the synthesis of the capsule polysaccharides, except for one gene that showed a frameshift mutation [30] and the overall biochemical structure as explained above. This could explain why both phages, πVLC5 and πVLC6, were able to infect these two capsular types. In principle, the depolymerase shared between these two phages (and the phages previously described by our group) should be responsible for degrading the exopolysaccharides shared between K22 and K37. The ability of the phage πVLC6 to also infect capsular type K13 and digest K2 capsule should be conferred by its second depolymerase, due to the divergence shown between the capsular biochemical structures. This second putative depolymerase presented high nucleotide identity (96% and 100% of coverage) with the second tail-fiber protein of bacteriophage *vB_KpnP_KpV74* [16]. Solovieva et al., cloned and expressed this protein (kpv74_56, APZ82768.1) and showed that it was a functional depolymerase against K2 strains and one K13 strain, thus, sharing not only the nucleotide identity but also host tropism with the second depolymerase of bacteriophage *πVLC6* [22]. The association between K2 and K13 capsule digestion was also supported by previous work showing cross-infectivity between the K2 and K13 types [31]. The additional interaction of phage *πVLC6* with the K3 strain was less expected, as the biochemical structure of this antigen overlapped only partially with K22/K37 and K2/K13, so both enzymes might be able to recognize the K3 antigen. However, there are no reported cases of cross-reaction involving these antigens. To our knowledge, the only depolymerase confirmed to have K3 tropism is that of the *Klebsiella* phage *KP32*, KP32gp37 [5]. Interestingly, this protein is the only one described so far that presents the identity of the enzymatic domain of the first tail spike shared by all *πVLC* phages (Table S1). Thus, the diversity of this enzymatic domain as well as the role of the second tail spike should be further explored, in order to explain K3 tropism. Lastly, we did not find any additional host specificity of *πVLC5*, despite the presence of a highly divergent depolymerase. Screening against additional novel capsular types, which are constantly being described [32], might reveal its role. 

In conclusion, the two new phages *πVLC5* and *πVLC6*, showed interesting aspects of the *Drulisvirus* evolution and suggest that host range expansion can occur via the acquisition of divergent depolymerases. Yet, few broad-range *Klebsiella* phages have been described so far. Further prospection of environmental samples might reveal new, more broadly-acting phages. In future work, it would also be interesting to explore resistance mechanisms, since in most cases bacterial growth fully relapses after few hours. In vitro resistance conferred by capsule loss might be inconsequential in vivo, since de-capsulated mutants tend to be avirulent [33]. Finally, testing synergistic effects between phages and antibiotics is yet another promising line of research [34,35]. 

## 4. Materials and Methods 

### 4.1. Bacteria

The clinical isolate 1210 obtained from Hospital La Fe (Valencia, Spain) was used as a host to test environmental samples for phage discovery. In addition, 77 reference strains of *Klebsiella* spp. purchased from the Statens Serum Institute (Copenhagen, Denmark; Table S3) were used to determine the range of infection of the isolated phages.

### 4.2. Isolation of Phages from Environmental Samples

A water sample obtained from a river and another from an irrigation ditch were used to isolate phages. For this purpose, 50 mL of water were taken and kept at room temperature until processing in the laboratory, typically 1 h later. After vortexing, samples were centrifuged (4000× *g*, 10 min) and supernatants filtered through 0.45 μm and 0.22 μm, to remove the cells and debris. Then, 1 mL of the filtered samples was added to 500 μL of *K. pneumoniae* clinical isolate 1210 and plated on Petri dishes, through the top agar method. Plates were incubated at 37 °C for 20 h. Isolated plaques of different morphology were then recovered by aspiration with a micropipette and stored at −70 °C. In order to check the isolated plaques and purify them, two additional plaque assays and plaque picking steps were performed. The plaque-purified phages were amplified in liquid media by infecting an exponential growth phase culture of the bacterium (OD_600_ = 0.4). After lysis, bacteria and debris were removed by centrifugation (13,000× *g*, 3 min twice) and the supernatants were aliquoted and stored at −70 °C. 

### 4.3. Electron Microscopy

High titer lysates were centrifuged and supernatants were filtered through 0.22 µm to remove bacteria and debris. A drop from each lysate was then deposited onto a carbon-coated Formvar supported by a 300 mesh copper grid. After air drying for 30 min, excess liquid was removed with filter paper. Phages were negatively stained with 2% phosphotungstic acid and examined under an electron microscope Jeol JEM-1010.

### 4.4. DNA Isolation and Genome Sequencing

DNA was extracted by treating high titer filtered lysates with DNAse I to remove non-encapsidated DNA and, following DNAse I inactivation, DNA extraction was performed using a commercial kit (QIAamp, QIAGEN). DNA was then fragmented by sonication and sequenced in an Illumina MiSeq machine yielding 250 paired-end reads, which were screened for contaminants using Kraken 2 [36] and then assembled with SPAdes v3.9.1 [37]. Contigs smaller than 1000 nucleotides were discarded, resulting in a single contig for each genome. PHASTER [38] and BLAST were used to determine the closest sequences in the databases. These were downloaded, and we used ProgressiveMAUVE [39] to obtain a whole-genome multiple sequence alignment. Complete genome sequences are available at Genbank (accession No. MT197175-MT197176).

### 4.5. Genome Annotation

Phages were annotated using PHANOTATE [40], Glimmer v.3.0 [41], and Prodigal [42]. Gene callings and start and stop coordinates were compared with a custom script available at https://github.com/BBeamud/. Nucleotide sequences for the putative ORFs were extracted with seqtk subseq (https://github.com/lh3/seqtk) and used for functional annotation. A phage nucleotide database was built with makeblastdb from the one available at http://millardlab.org/bioinformatics/bacteriophage-genomes/. This database contains all non-redundant phage proteins sequences deposited in GenBank and their annotation (August, 2019). Phages *πVLC* were added to this database [18]. The best BLASTN hit of non-hypothetical proteins, when possible, was retrieved with an e-value cutoff of 10^−5^. We also searched for temperate behavior in our phage genomes, such as mobile genetic elements, antibiotic resistance or virulence genes, and any kind of bacterial gene. Local databases from ICEberg v.2.0 (http://db-mml.sjtu.edu.cn/ICEberg/), the Comprehensive Antibiotic Resistance Database (CARD, https://card.mcmaster.ca/), Virulence factors of Pathogenic Bacteria (VFDB, http://www.mgc.ac.cn/VFs/main.htm), and BacteriaDB (in-house database, 2016) were used to accomplish this using BLASTN, with an e-value cutoff of 10^−5^. Lastly, CDSs were searched against a local database constructed with depolymerase proteins included in Pires et al. [43]. InterProScan5 [44] and HHpred [45] were used for further checking of domains and protein structures when a significant similarity was found. 

### 4.6. Comparative Genomics

Whole-genome average nucleotide identity (ANI) values between the new *Klebsiella* phages and the other *Drulisvirus* phages were obtained using pANIto (https://github.com/sanger-pathogens/panito). Conserved and unique genes among the *Drulisvirus* were obtained with Proteinortho v.6 [46], by comparing the previously extracted nucleotide CDSs and the ones available from GenBank. Maximum likelihood (ML) phylogenies were constructed with 1000 fast bootstrap pseudo-replicates using the GTR+G+I substitution model in IQ-TREE v.1.6.5 [47]. 

### 4.7. Determination of Host Range

Spot tests were performed by adding 2 µL drops of each high titer phage to the bacterial lawns of the 77 reference *Klebsiella* spp. strains in top-agar semi-solidified media, and incubating plates at 37 °C for 24 h. In addition, we inoculated cultures of the 77 reference strains in w-96 plates with 10^5^ PFU of phage and incubated cultures at 37 °C in a plate reader (Multiskan) for 10 h, to determine the strength of lysis based OD measurements taken every 10 min. 

### 4.8. Determination of Phage Progeny Production

Approximately 10^5^ PFU were used to inoculate 500 µL of log-phase bacterial cultures (OD_600_ = 0.05) in a Thermoshaker, and the cultures were incubated at 250 rpm and 37 °C. At 4 hpi, the cultures were centrifuged (13,000× g, 2 min) to remove bacteria and debris, and the supernatants were used for titration. To accomplish this, the supernatants were serially diluted in LB, and then used to perform plaque assays in 1210 *K. pneumoniae* cultures. A similar approach was used to determine phage progeny production in semi-solid cultures. Spots with ca. 10^5^ PFU were used to inoculate bacterial cultures, that were incubated at 37 °C for 8 h. After this, a micropipette was used to recover the phages from the spot, which were resuspended in LB for titration. 

### 4.9. Capsule Light Microscopy

Bacteria were grown in semi-solidified media in petri dishes to perform spot tests with phage suspensions (≥10^7^ PFU/mL). After 24 h at 37 °C, a smear loop was used to sample bacteria from the haloes and non-inoculated regions of the plate. Samples were fixed with 2.5% formaldehyde for 20 min, in the presence of 100 mM lysine, which prevented capsule loss during fixation [48]. After removing the fixative agent through centrifugation and washing with low-salt TE buffer, 10% nigrosin was mixed with the bacterial suspension on a glass slide and 1% crystal violet was added. The preparations were examined through a light microscopy and the presence of capsules was assessed by the exclusion of both nigrosin and crystal violet around the cells. 

### 4.10. Structural Comparison of K Antigens

To determine if the capsular K antigens that showed any type of interaction with the novel phages were structurally similar, we downloaded the atomic structure of each antigen from https://iith.ac.in/K-PAM/k_antigen.html [49]. Program database (PDB) files were loaded and aligned with the PyMOL Molecular Graphics System (Version 2.0 Schrödinger, LLC). The ‘align’ command performed a structural superposition with several rounds of refinement to discard structural outliers. We obtained the number of aligned atoms and the root-mean-square deviation of atomic positions (RMSD) between the pairwise-alignments of K antigens.

## Figures and Tables

**Table 1:** Depolymerase motifs identified in *Klebsiella* phages *πVLC5* and *πVLC6*.

Phage	ORF	Protein Size (aa)	Tool	Motif (aa)	Family	Identifier	e-Value
***πVLC5***	ORF49 (36103–38478)	792	InterProScan5	13–140	Bacteriophage *T7* tail fiber protein	IPR005604	5.1 × 10^−14^
				319–617	Pectin lyase	IPR012334	3.2 × 10^−10^
			HHpred	2–578	*phiAB6* tail spike	5JSD_B	2.1 × 10^−23^
				234–789	Putative tail fiber; Tail spike, hydrolase	5W6S_A	2.3 × 10^−13^
	ORF58 (42631–44634)	668	InterProScan5	16–365	Pectin lyase	IPR012334	1.0 × 10^−15^
				544–603	Chaperone of endosialidase	IPR030392	7.3 × 10^−37^
				544–667	Winged helix-like DNA-binding domain	IPR036388	5.8 × 10^−6^
			HHpred	26–665	Tail spike protein *Acinetobacter* phage	6EU4_B	6.0 × 10^−34^
***πVLC6***	ORF51 (35765–38140)	792	InterProScan5	13–140	Bacteriophage *T7* tail fiber protein	IPR005604	1.7 × 10^−14^
				319–617	Pectin lyase	IPR012334	9.1 × 10^−10^
			HHpred	1–578	*phiAB6* tail spike	5JSD_B	6.3 × 10^−23^
	ORF58 (42292–44025)	578	HHpred	1–576	Bacteriophage *CBA120* tail spike	6EU4_B	2.1 × 10^−41^

## Supplementary Materials

The following are available online at https://www.mdpi.com/1422-0067/21/9/3160/s1. Table S1: Results of the BLASTN search of the tail fiber sequences for phages *πVLC5* and *πVLC6* against a database of phage CDSs. Table S2: Determination of *Klebsiella* phages *πVLC5* and *πVLC6* host range in semi-solid and liquid. Table S3: *Klebsiella* species and K-serotypes of the 77 K-type reference strains from the Statens Serum Institute (Copenhagen, Denmark).
