# Supplementary material for: Isolation and Characterization of Two Klebsiella pneumoniae Phages Encoding Divergent Depolymerases
Source: Int J Mol Sci. 2020 Apr 30;21(9):3160. doi: 10.3390/ijms21093160 (PMC7246685; doi:10.3390/ijms21093160)
Supplement: Supplementary file 1 [file ijms-21-03160-s001.zip › ijms-768073-for publication-supplementary/Table S1 final revised.docx]

**Table S1. Results of the BLASTN search for tail fiber/spike sequences using a database of phage CDSs.**

| **Query** | **Subject (phage name, protein ID, taxonomy*)** | | | **Subject Length** | **Alignment Length** | **Query Start** | **Query End** | **Subject Start** | **Subject End** | **Hsp Expect** | **Identity** | **Query cover** |
| --- | --- | --- | --- | --- | --- | --- | --- | --- | --- | --- | --- | --- |
| **ORF49**  ***K*. phage *πVLC5*** | *Klebsiella* phage *πVLC6* | ORF51 | *Drulisvirus* | 2376 | 2376 | 1 | 2376 | 1 | 2376 | 0 | 0.96 | 1.00 |
|  | *Klebsiella* phage *πVLC4* | QGZ00936.1 | *Drulisvirus* | 2235 | 2237 | 1 | 2237 | 1 | 2204 | 0 | 0.90 | 0.94 |
|  | *Klebsiella* phage *πVLC2* | QGZ00819.1 | *Drulisvirus* | 2343 | 2376 | 1 | 2376 | 1 | 2343 | 0 | 0.90 | 1.00 |
|  | *Klebsiella* phage *πVLC1* | QGZ00758.1 | *Drulisvirus* | 2235 | 2237 | 1 | 2237 | 1 | 2204 | 0 | 0.90 | 0.94 |
|  | *Klebsiella* phage *πVLC3* | QGZ00875.1 | *Drulisvirus* | 2343 | 2376 | 1 | 2376 | 1 | 2343 | 0 | 0.90 | 1.00 |
|  | *Klebsiella* phage *KpV41* | YP_009188788.1 | *Drulisvirus* | 2577 | 798 | 1 | 798 | 1 | 798 | 0 | 0.92 | 0.34 |
|  | *Klebsiella* phage *vB_KpnP_SU552A* | YP_009204835.1 | *Drulisvirus* | 2382 | 785 | 1 | 785 | 1 | 785 | 0 | 0.92 | 0.33 |
|  | *Klebsiella* phage *phiKpS2* | AWK24039.1 | *Drulisvirus* | 2841 | 773 | 1 | 773 | 1 | 773 | 0 | 0.89 | 0.33 |
|  | *Klebsiella* phage *vB_KpnP_KpV74* | APZ82760.1 | *Drulisvirus* | 1809 | 429 | 1 | 429 | 1 | 429 | 2.36E-159 | 0.89 | 0.18 |
|  | *Klebsiella* phage *KpV475* | YP_009280712.1 | *Drulisvirus* | 957 | 526 | 1 | 525 | 1 | 525 | 4.01E-93 | 0.75 | 0.22 |
|  | *Klebsiella* phage *phiBO1E* | AIT13620.1 | *Drulisvirus* | 957 | 695 | 1 | 686 | 1 | 689 | 2.08E-90 | 0.71 | 0.29 |
|  | *Klebsiella* phage *KPV811* | APD20665.1 | *Drulisvirus* | 957 | 526 | 1 | 525 | 957 | 433 | 1.08E-87 | 0.74 | 0.22 |
|  | *Klebsiella* phage *vB_KpnP_KpV48* | AOZ65257.1 | *Drulisvirus* | 957 | 690 | 1 | 686 | 1 | 689 | 5.57E-85 | 0.71 | 0.29 |
|  | *Klebsiella* phage *F19* | YP_009006065.2 | *Drulisvirus* | 924 | 468 | 1 | 468 | 1 | 465 | 2.89E-82 | 0.75 | 0.20 |
|  | *Klebsiella* phage *NTUH-K2044-K1-1* | YP_009098379.1 | *Drulisvirus* | 957 | 679 | 1 | 676 | 1 | 679 | 3.52E-81 | 0.70 | 0.29 |
|  | *Klebsiella* phage *KpV71* | YP_009302749.1 | *Drulisvirus* | 957 | 679 | 1 | 676 | 1 | 679 | 3.52E-81 | 0.70 | 0.29 |
|  | *Klebsiella* phage *vB_KpnP_SU503* | YP_009199929.1 | *Drulisvirus* | 924 | 459 | 1 | 459 | 1 | 459 | 1.71E-72 | 0.73 | 0.19 |
|  | *Escherichia* phage *Minorna* | QBP07062.1 | *Drulisvirus* | 753 | 368 | 1 | 368 | 753 | 386 | 5.95E-72 | 0.77 | 0.15 |
|  | *Klebsiella* phage *Kp2* | YP_009188359.1 | *Drulisvirus* | 1593 | 482 | 2 | 481 | 2 | 481 | 8.84E-70 | 0.73 | 0.20 |
|  | *Klebsiella* phage *AltoGao* | ASV44938.1 | *Drulisvirus* | 924 | 433 | 1 | 431 | 1 | 431 | 1.08E-68 | 0.74 | 0.18 |
|  | *Shigella* phage *SFN6B* | AVD98997.1 | *Drulisvirus* | 1047 | 478 | 13 | 487 | 70 | 523 | 3.76E-68 | 0.72 | 0.20 |
|  | *Klebsiella* phage *KP34* | YP_003347643.1 | *Drulisvirus* | 924 | 459 | 1 | 459 | 1 | 459 | 1.95E-65 | 0.72 | 0.19 |
|  | *Enterobacter* phage *phiKDA1* | AFE86146.1 | *Autographivirinae* | 1581 | 312 | 61 | 369 | 133 | 441 | 1.83E-21 | 0.68 | 0.13 |
|  | *Pantoea* phage *LIMElight* | CBW54806.1 | *Phikmvvirus* | 2565 | 153 | 268 | 419 | 265 | 416 | 1.4E-16 | 0.75 | 0.06 |
|  | *Shigella* phage *HRP29* | QBP32944.1 | *Drulisvirus* | 783 | 227 | 181 | 407 | 187 | 413 | 1.71E-15 | 0.69 | 0.10 |
|  | *Klebsiella* virus *KP32* | YP_003347555.1 | *Przondovirus* | 2334 | 121 | 1423 | 1543 | 1402 | 1522 | 8.30E-07 | 0.71 | 0.05 |
| **ORF58**  ***K*. phage *πVLC5*** | *Klebsiella* phage *KOX1* | ARM70347.1 | *Webervirus* | 2298 | 1023 | 218 | 1229 | 1559 | 548 | 3.16E-87 | 0.68 | 0.51 |
|  | *Klebsiella* phage *NJS1* | AXF39389.1 | *Webervirus* | 2979 | 267 | 1600 | 1865 | 432 | 173 | 3.17E-11 | 0.67 | 0.13 |
|  | *Vibrio* phage *1.164.O._10N.261.51.A7* | AUR91777.1 | *Unclassified* | 2430 | 44 | 1819 | 1862 | 2242 | 2285 | 8.51E-06 | 0.89 | 0.02 |
| **ORF58**  ***K.* phage *πVLC6*** | *Klebsiella* phage *phiKpS2* | AWK24047.1 | *Drulisvirus* | 1746 | 1746 | 1 | 1734 | 1 | 1746 | 0 | 0.96 | 1.01 |
|  | *Klebsiella* phage *F19* | YP_009006074.1 | *Drulisvirus* | 1734 | 1734 | 1 | 1734 | 1 | 1734 | 0 | 0.96 | 1.00 |
|  | *Klebsiella* phage *vB_KpnP_KpV74* | APZ82768.1 | *Drulisvirus* | 1734 | 1734 | 1 | 1734 | 1 | 1734 | 0 | 0.95 | 1.00 |
