# Supplementary material for: Isolation and Characterization of Two Klebsiella pneumoniae Phages Encoding Divergent Depolymerases
Source: Int J Mol Sci. 2020 Apr 30;21(9):3160. doi: 10.3390/ijms21093160 (PMC7246685; doi:10.3390/ijms21093160)
Supplement: Supplementary file 1 [file ijms-21-03160-s001.zip › ijms-768073-for publication-supplementary/Table S2 final revised.docx]

**Table S2. Determination of *Klebsiella* phages *πVLC5* and *πVLC6* host range in semi-solid and liquid media**.

| **K-type** | ***Klebsiella phage πVLC5*** | | ***Klebsiella phage πVLC6*** | |
| --- | --- | --- | --- | --- |
|  | **Spot test** | **Liquid infection** | **Spot test** | **Liquid infection** |
| 1 | *-* | *-* | - | *-* |
| 2 | *-* | *-* | *+* | *-* |
| 3 | *-* | *-* | *+* | *-* |
| 4 | *-* | *-* | *-* | *-* |
| 5 | *-* | *-* | *-* | *-* |
| 6 | *-* | *-* | *-* | *-* |
| 7 | *-* | *-* | *-* | *-* |
| 8 | *-* | *-* | *-* | *-* |
| 9 | *-* | *-* | *-* | *-* |
| 10 | *-* | *-* | *-* | *-* |
| 11 | *-* | *-* | *-* | *-* |
| 12 | *-* | *-* | *-* | *-* |
| 13 | *-* | *-* | *++* | *++* |
| 14 | *-* | *-* | *-* | *-* |
| 15 | *-* | *-* | *-* | *-* |
| 16 | *-* | *-* | *-* | *-* |
| 17 | *-* | *-* | *-* | *-* |
| 18 | *-* | *-* | *-* | *-* |
| 19 | *-* | *-* | *-* | *-* |
| 20 | *-* | *-* | *-* | *-* |
| 21 | *-* | *-* | *-* | *-* |
| 22 | *++* | *++* | *++* | *++* |
| 23 | *-* | *-* | *-* | *-* |
| 24 | *-* | *-* | *-* | *-* |
| 25 | *-* | *-* | *-* | *-* |
| 26 | *-* | *-* | *-* | *-* |
| 27 | *-* | *-* | *-* | *-* |
| 28 | *-* | *-* | *-* | *-* |
| 29 | *-* | *-* | *-* | *-* |
| 30 | *-* | *-* | *-* | *-* |
| 31 | *-* | *-* | *-* | *-* |
| 32 | *-* | *-* | *-* | *-* |
| 33 | *-* | *-* | *-* | *-* |
| 34 | *-* | *-* | *-* | *-* |
| 35 | *-* | *-* | *-* | *-* |
| 36 | *-* | *-* | *-* | *-* |
| 37 | *++* | *++* | *++* | *++* |
| 38 | *-* | *-* | *-* | *-* |
| 39 | *-* | *-* | *-* | *-* |
| 40 | *-* | *-* | *-* | *-* |
| 41 | *-* | *-* | *-* | *-* |
| 42 | *-* | *-* | *-* | *-* |
| 43 | *-* | *-* | *-* | *-* |
| 44 | *-* | *-* | *-* | *-* |
| 45 | *-* | *-* | *-* | *-* |
| 46 | *-* | *-* | *-* | *-* |
| 47 | *-* | *-* | *-* | *-* |
| 48 | *-* | *-* | *-* | *-* |
| 49 | *-* | *-* | *-* | *-* |
| 50 | *-* | *-* | *-* | *-* |
| 51 | *-* | *-* | *-* | *-* |
| 52 | *-* | *-* | *-* | *-* |
| 53 | *-* | *-* | *-* | *-* |
| 54 | *-* | *-* | *-* | *-* |
| 55 | *-* | *-* | *-* | *-* |
| 56 | *-* | *-* | *-* | *-* |
| 57 | *-* | *-* | *-* | *-* |
| 58 | *-* | *-* | *-* | *-* |
| 59 | *-* | *-* | *-* | *-* |
| 60 | *-* | *-* | *-* | *-* |
| 61 | *-* | *-* | *-* | *-* |
| 62 | *-* | *-* | *-* | *-* |
| 63 | *-* | *-* | *-* | *-* |
| 64 | *-* | *-* | *-* | *-* |
| 65 | *-* | *-* | *-* | *-* |
| 66 | *-* | *-* | *-* | *-* |
| 67 | *-* | *-* | *-* | *-* |
| 68 | *-* | *-* | *-* | *-* |
| 69 | *-* | *-* | *-* | *-* |
| 70 | *-* | *-* | *-* | *-* |
| 71 | *-* | *-* | *-* | *-* |
| 72 | *-* | *-* | *-* | *-* |
| 74 | *-* | *-* | *-* | *-* |
| 79 | *-* | *-* | *-* | *-* |
| 80 | *-* | *-* | *-* | *-* |
| 81 | *-* | *-* | *-* | *-* |
| 82 | *-* | *-* | *-* | *-* |

+: weak infectivity or halo-like spot

++: strong infectivity

-: non infected
