# Supplementary material for: Isolation and Characterization of Two Klebsiella pneumoniae Phages Encoding Divergent Depolymerases
Source: Int J Mol Sci. 2020 Apr 30;21(9):3160. doi: 10.3390/ijms21093160 (PMC7246685; doi:10.3390/ijms21093160)
Supplement: Supplementary file 1 [file ijms-21-03160-s001.zip › ijms-768073-for publication-supplementary/Table S3 final revised.docx]

**Table S3. *Klebsiella* species and K-serotypes of the 77 K-type reference strains from the Statens Serum Institute (Copenhagen, Denmark).**

| **K-type** | **Species** | **Strain number** |
| --- | --- | --- |
| 1 | *K. pneumoniae* | A 5054 |
| 2 | *K. pneumoniae* | B 5055 |
| 3 | *K. pneumoniae* | C 5046 |
| 4 | *K. ozaenae* | D 5050 |
| 5 | *K. ozaenae* | E 5051 |
| 6 | *K. pneumoniae* | F 5052 |
| 7 | *K. pneumoniae* | Aerogenes 4140 |
| 8 | *K. planticola* | Klebs. 1015 |
| 9 | *K. pneumoniae* | Klebs. 56 |
| 10 | *K. pneumoniae* | Klebs. 919 |
| 11 | *K. pneumoniae* | Klebs. 390 |
| 12 | *K. pneumoniae* | Klebs. 313 |
| 13 | *K. pneumoniae* | Klebs. 1470 |
| 14 | *K. planticola* | Klebs. 1193 |
| 15 | *K. pneumoniae* | Mich. 61 |
| 16 | *K. pneumoniae* | 2069/49 |
| 17 | *K. pneumoniae* | 2005/49 |
| 18 | *K. pneumoniae* | 1754/49 |
| 19 | *K. pneumoniae* | 293/50 |
| 20 | *K. pneumoniae* | 889/50 |
| 21 | *K. pneumoniae* | 1702/49 |
| 22 | *K. pneumoniae* | 1996/49 |
| 23 | *K. pneumoniae* | 2812/50 |
| 24 | *K. pneumoniae* | 1680/49 |
| 25 | *K. pneumoniae* | 2002/49 |
| 26 | *K. oxytoca* | 5884 |
| 27 | *K. pneumoniae* | 6613 |
| 28 | *K. pneumoniae* | 5758 |
| 29 | *K. oxytoca* | 5725y |
| 30 | *K. pneumoniae* | 7824 |
| 31 | *K. pneumoniae* | 6258 |
| 32 | *K. planticola* | 6837 |
| 33 | *K. pneumoniae* | 6168 |
| 34 | *K. pneumoniae* | 7522 |
| 35 | *K. planticola* | 7444 |
| 36 | *K. pneumoniae* | 8306 |
| 37 | *K. pneumoniae* | 8238 |
| 38 | *K. pneumoniae* | 8414 |
| 39 | *K. planticola* | 7749 |
| 40 | *K. pneumoniae* | 8588 |
| 41 | *K. oxytoca* | 6177 |
| 42 | *K. pneumoniae* | 1702 |
| 43 | *K. pneumoniae* | 2482 |
| 44 | *K. planticola* | 7730 |
| 45 | *K. pneumoniae* | 8464 |
| 46 | *K. pneumoniae* | 5281 |
| 47 | *K. pneumoniae* | 9682 |
| 48 | *K. planticola* | 1196 |
| 49 | *K. planticola* | 6115 |
| 50 | *K. pneumoniae* | 1303/50 |
| 51 | *K. pneumoniae* | 4715/50 |
| 52 | *K. pneumoniae* | 5759/50 |
| 53 | *K. planticola* | 1756/51 |
| 54 | *K. planticola* | Stanley |
| 55 | *K. pneumoniae* | 3985/51 |
| 56 | *K. planticola* | 3534/51 |
| 57 | *K. planticola* | 4425/51 |
| 58 | *K. planticola* | 636/52 |
| 59 | *K. planticola* | 2212/52 |
| 60 | *K. pneumoniae* | 4463/52 |
| 61 | *K. pneumoniae* | 5710/52 |
| 62 | *K. pneumoniae* | 5711/52 |
| 63 | *K. pneumoniae* | 5845/52 |
| 64 | *K. pneumoniae* | NCTC 8172 |
| 65 | *K. terrigena* | SW 4 |
| 66 | *K. oxytoca* | 438 (3a) |
| 67 | *K. pneumoniae* | 264 (1) |
| 68 | *K. pneumoniae* | 265 (1) |
| 69 | *K. pneumoniae* | 889 |
| 70 | *K. oxytoca* | 167 |
| 71 | *K. planticola* | 4349 |
| 72 | *K. planticola* | 1205 |
| 74 | *K. oxytoca* | 371 |
| 79 | *K. planticola* | 325 |
| 80 | *K. pneumoniae* | 708 |
| 81 | *K. pneumoniae* | 370 |
| 82 | *K. pneumoniae* | 3454-70 |
